# Advancing Use of DEXA Scans to Quantitatively and Qualitatively Evaluate Lateral Spinal Curves, for Preliminary Identification of Adolescent Idiopathic Scoliosis

**DOI:** 10.1007/s00223-023-01075-2

**Published:** 2023-03-13

**Authors:** P. T. T. Ng, L. Straker, K. Tucker, M. T. Izatt, A. Claus

**Affiliations:** 1 Laboratory for Motor Control and Pain Research, School of Biomedical Sciences, The University of Queensland, St. Lucia, Brisbane, QLD Australia; 2 Physiotherapy Department, KK Women’s and Children’s Hospital, Singapore, Singapore; 3 School of Allied Health, Curtin University, Bentley, Perth, WA Australia; 4 Biomechanics and Spine Research Group, Queensland University of Technology at the Centre for Children’s Health Research, South Brisbane, Brisbane, QLD Australia; 5 School of Health & Rehabilitation Sciences, The University of Queensland, St. Lucia, Brisbane, QLD Australia; 6 Tess Cramond Pain and Research Centre, Royal Brisbane and Women’s Hospital, Herston, Brisbane, QLD Australia

**Keywords:** Adolescent idiopathic scoliosis, DEXA, Raine Study, Modified Ferguson method, Prevalence

## Abstract

Dual-energy X-ray absorptiometry (DEXA) scan is an emerging screening method for identifying likely adolescent idiopathic scoliosis (AIS). Using DEXA in an unbiased population sample (the Raine Study), we aimed to report the inter-rater reliability and minimal detectable change (MDC_95_) for scoliosis curve angle measurement, identify likely AIS prevalence, and the concordance between reported AIS diagnosis and DEXA-identified likely AIS. Scoliosis curve angles were measured using the modified Ferguson method on DEXA scans (*n* = 1238) at age 20 years. For curve angle inter-rater reliability, two examiners measured angles (6–40°) on 41 scans. Likely, AIS was determined with quantitative and qualitative criteria (modified Ferguson angles ≥ 10° and expert review of spinal curves).The inter-rater reliability for scoliosis curve angle measurement was good–excellent (ICC: 0.82; 95% CI: 0.71–0.89; *p* < 0.001), and MDC_95_ was 6.2°. The prevalence of likely AIS was 2.1% (26/1238). Diagnosis of AIS was reported despite little or no scoliosis curve (< 3.8°) for 20 participants (1.6%), and diagnosis of AIS was not reported despite scoliosis curve ≥ 10° for 11 participants (0.9%). Results support the use of modified Ferguson method to measure scoliosis curve angles on DEXA. There is potential utility for using a combination of quantitative measurement and qualitative criteria to evaluate DEXA images, to identify likely AIS for reporting prevalence. Without formal school screening, the analysis of DEXA in this population sample suggested that relying on current health professional diagnosis alone could result in 2.5% of this cohort being at risk of false positive diagnosis or delay in necessary management due to non-diagnosis of AIS.

**Supplementary Information:**

The online version contains supplementary material available at 10.1007/s00223-023-01075-2.

## Introduction

Adolescent idiopathic scoliosis (AIS) is a three-dimensional deformity of the spine that is diagnosed between the ages of 10 to 18 years. Diagnosis of AIS requires exclusion of other causes of spinal deformity and a ≥ 10° Cobb angle measurement of the scoliosis curve on a standing radiograph. AIS is most commonly reported to be present in 2–3% of the adolescent population worldwide [1]. Between the ages of 11 to 14 years old, adolescents experience rapid growth, which is associated with rapid progression of curves in those with scoliosis [2]. Approximately, 15% of adolescents with AIS progress to instrumented spinal fusion surgery [3]. Globally, methods of AIS identification in the community range from incidental finding of spinal curve during health professional’s usual care to formal adolescent screening programs in schools. In order to examine the ability of these methods to identify AIS, there is also a need for unbiased methods of sampling the population.

Formal school screening programs for scoliosis are usually performed for adolescents aged 11–14 years [4], and the most commonly used method for screening is scoliometer measurement of the rib hump in Adam’s forward bending test [5–7]. Other methods to identify AIS include public health awareness campaigns that direct individuals to attend a healthcare setting if AIS is suspected [8] or health professionals’ identification of AIS during ad hoc clinic visits [9]. To compare the rates of scoliosis identification, and relative health implications with and without formal screening, a study from Norway compared two time periods with differing methods [9]. They reported earlier detection of AIS with a formal screening program than with health professional identification during ad hoc clinic visits that resulted in a higher number of adolescents with AIS conservatively managed with spinal bracing (41/year with screening vs 20/year without screening) and a lower number requiring spinal fusion surgery (19/year with screening vs 32/year without screening) [9]. However, observational study methods, such as that used in this Norwegian study, include considerable risk from confounding variables, including generational, spatial (clinic location) and management pathway differences between the cohorts compared. To progress understanding of the health outcomes resulting from the different methods of identifying AIS, it may also be useful to consider data from an unbiased sample of the population.

An unbiased population sample is presented by the Raine Study, a prospective longitudinal cohort study with data collection on a broad range of health measures [10]. The Raine Study recruited antenatal women and their offspring in Perth, Australia, between 1989 and 1992, and dual-energy X-ray absorptiometry (DEXA) scans were collected when the offspring were 20 years old, to measure adiposity, muscle mass, and bone density. The scan data present an opportunity to advance the methods previously developed for analysis of scoliosis curves on DEXA and to identify those that likely have AIS.

DEXA is an emerging option for spinal screening to identify likely the presence of AIS and estimation of the population prevalence from the anteroposterior view of the spine in supine lying. To identify and measure scoliosis with DEXA scans, a modified Ferguson method was developed by Taylor et al. [11] in the Avon Longitudinal Study of Parents and Children (ALSPAC). That study evaluated the precision of the modified Ferguson method. Measurements repeated by two raters showed that 95% of modified Ferguson angles were within 5° with an inter-rater difference mean (range) in angle of 2 (0–6)°. Comparison of these measurements with the “gold standard” Cobb angle on standing radiograph method showed that the modified Ferguson curve angles on supine DEXA scans resulted in ~ 39% smaller angles [11]. While Taylor et al. [11] determined the inter-rater reliability of the identification of likely scoliosis on DEXA scans (κ 0.74), the inter-rater reliability of the modified Ferguson angle as determined on DEXA has not been established. The study also conducted a qualitative review to identify likely scoliosis on DEXA scans; however, they mainly considered positioning errors that may cause a spinal curve in the scanners [11]. Other issues such as internal shadowing and scoliosis other than idiopathic types were not highlighted.

In a sample from the Raine Study, the aims of this study were: (i) to report the inter-rater reliability and minimal detectable change (MDC_95_) for scoliosis curve measurement on DEXA scans, (ii) to identify likely AIS and report its prevalence, using a combination of quantitative (modified Ferguson angle of ≥ 10°) and qualitative DEXA examination (expert review of images), and (iii) explore the concordance between health professional diagnosis of AIS with likely AIS identified via screening of the DEXA scans.

## Methods

This study used data from the Raine Study (www.rainestudy.org.au). This longitudinal cohort recruited 2868 mothers (generation one) and their children (generation two) born between 1989 and 1991, with prospective collection of a wide array of biological, psychological, and social variables. The Raine Study has been demonstrated to be an unbiased sample, representing the general population in Western Australia [10]. This study draws on DEXA scans collected at age 20 years (2009–2012), and International Classification of Diseases, Ninth Revision (ICD-9), code number 737.3 (idiopathic scoliosis/kyphoscoliosis) data collected at ages 1–20 years. This study was approved by the Raine Study Scientific Management Committee, ethics approval was obtained from the Human Research Ethics Committee of The University of Western Australia, and participants provided their own informed consent.

Participants who attended for a DEXA scan at age 20 years were included (*n* = 1238, females = 604, males = 634). The Norland XR-36 densitometer (Norland Medical Systems, Inc., Fort Atkinson, WA, USA) provided images of 96dpi with an image size of 280 × 730pixels, to measure lean and total fat mass. The research staff guided the participant’s upper and lower body to be in line with the pelvis while lying supine on the scanning table. Owing to skeletal maturity being likely at age 20, any scoliotic curves identified were expected to be at their maximum deformity (curve progression which is known to occur during skeletal growth has ceased) [12].

### Quantitative Measurement of Spinal Curve from DEXA Images

**Fig. 1:**
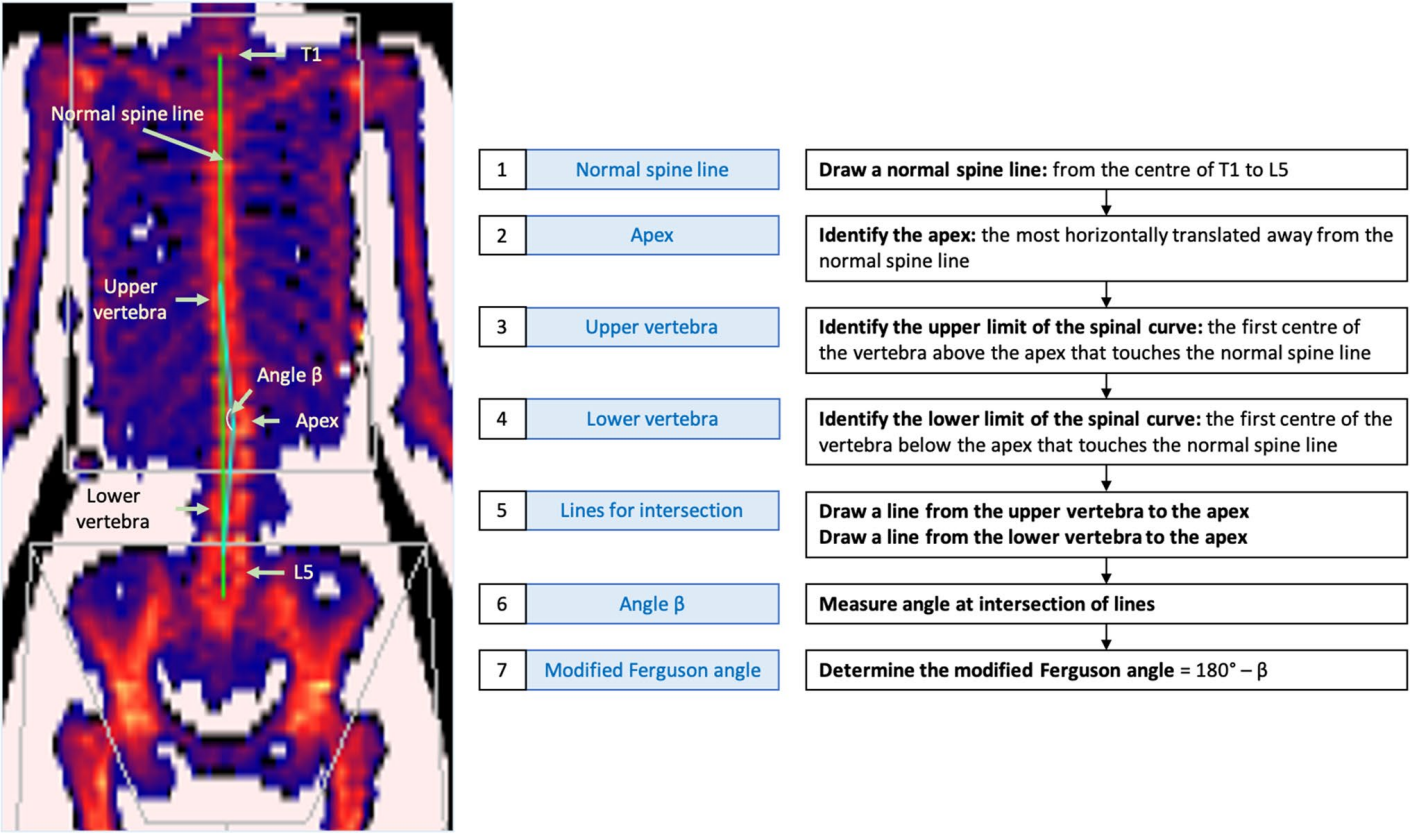
A DEXA scan with lines drawn for the modified Ferguson angle, with a flowchart describing the process (steps 1–7). This image shows symmetrical positioning with no obliquity of the shoulders or pelvis, and the lateral borders of the spine are clear to see without any cardiac shadowing

The low resolution of DEXA scan images means that it is not considered a diagnostic tool for identifying AIS. Formal diagnosis of AIS with radiographs or other advanced imaging typically employs either the Cobb method, which takes reference from the most inclined vertebral end plates of the major curve [13], or the Ferguson method, which takes reference from axial rotation of segments [14]. For preliminary identification and measurement of scoliosis on DEXA scans, the modified Ferguson method uses a “normal spine line” through the T1 to L5 vertebrae as a reference to determine the center of the upper and lower end vertebrae of a scoliotic curve, with lines connecting to the apex of the curve creating the measured angle [11]. Employing the modified Ferguson method in the current study, DEXA images were viewed at 300–400% magnification and marked with an image processing application (Pixelmator, version 3.3, Lithuania), as detailed in Fig. 1. At the intersection of the lines connecting the upper vertebra to apex and the lower vertebra to the apex, the angle β was measured with the ImageJ (version 2.0.0, National Institute of Health, USA) angle tool. The modified Ferguson angle was calculated by 180°- β. One author (AC) marked and measured all scans.

### Inter-Rater Reliability of DEXA Ferguson Method

To test the inter-rater reliability of marking and measuring these angles, a sample of 41 unmarked original DEXA scans, with angles ranging between 6° and 40° (as measured by examiner AC), were independently marked and measured by a second examiner (PN), who was blind to the previous measurements.

### Qualitative Examination of Scoliosis Curve

An apparent curve on a DEXA scan may represent poor participant positioning for the scan, a shadow effect from internal organs, a spinal pathology other than AIS, or spinal curves consistent with AIS. Due to these potential confounders, a clinical research expert with 25 years of experience and publications in relation to AIS image analysis and clinical deformity measurement (author-MI) [15–17] conducted a qualitative evaluation of DEXA scans that had modified Ferguson angles ≥ 8° (*n* = 142). The criteria that the expert applied to qualitatively evaluate scans are provided in Table 1, and exemplar images are provided in Online Appendix.

**Table 1:** Qualitative criteria used by the expert reviewer for evaluation of spinal curves on DEXA images, with the aim to identify likely adolescent idiopathic scoliosis

Description	Identifying features used
Positional error	Obliquity of the pelvis that may produce an isolated lumbar curve
	Obliquity of the shoulders that may produce an isolated cervical or upper thoracic curve
	Participant generally not lying straight on scan, seen with lateral translation of the shoulders relative to the pelvis
Image shadow from internal organs	Unequal width of vertebrae in the mid-thoracic spine with the absence of any sort of compensatory curve above and/or below the pseudo-scoliosis, thought to be from an aortic arch and/or cardiac shadow
Other spinal pathology (non-idiopathic)	Acutely angulated spine over 2–4 spinal segments suggestive of scoliosis not of idiopathic type or long C-shaped curve of spine suggestive of neuromuscular scoliosis
Consistent with idiopathic scoliosis	After exclusion of the above features, participant has a lateral curve involving > 4 spinal segments, in one of the common idiopathic scoliosis curve patterns (single thoracic/ thoracolumbar/ lumbar or balanced thoracic and lumbar curves)

### Reported Diagnosis of Idiopathic Scoliosis/Kyphoscoliosis

At ages 1, 5, 8, 10, 14, 17, and 20 years, participants/parents of the Raine Study reported on their health conditions that had been diagnosed by a health professional, based on the question: “Does your child have now, or has your child had in the past, any of the following health professional diagnosed medical conditions or health problems?”. These reports were subsequently classified by Raine Study research staff using the ICD-9 [18]. Those who had DEXA scans at age 20 and reported a diagnosis of “idiopathic scoliosis/kyphoscoliosis” at ages 1, 5, and 8 years (*n* = 2) were excluded from the dataset for likely AIS owing to juvenile-onset rather than adolescent-onset of their condition.

### Participant Grouping

**Fig. 2:**
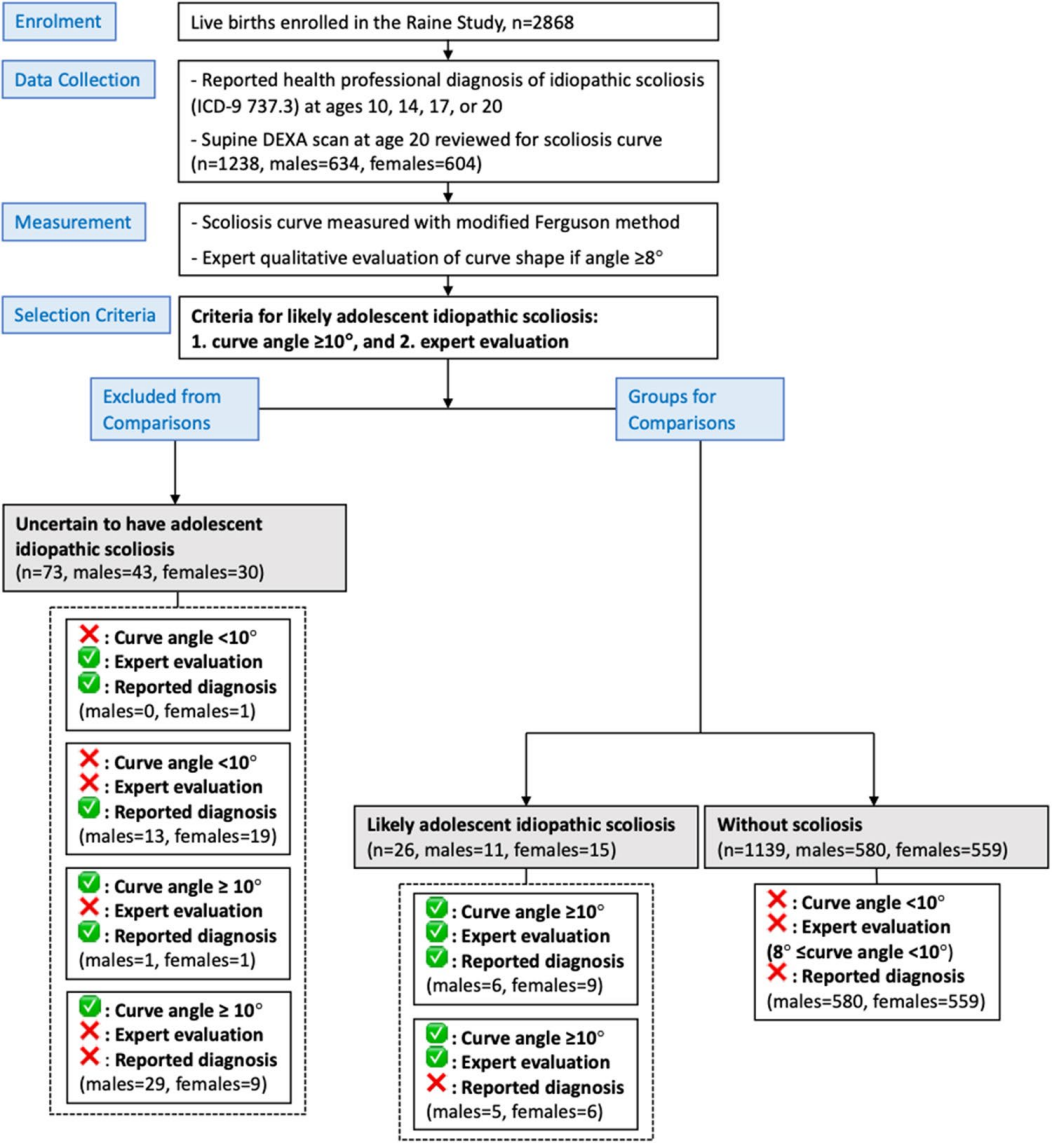
Consolidated standards of reporting trials (CONSORT) diagram to detail the recruitment and allocation of participants for this study. Participants were allocated into: (i) *likely adolescent idiopathic scoliosis,* (ii) *without scoliosis,* and *(iii) uncertain to have adolescent idiopathic scoliosis* based on their curve angle (measured modified Ferguson angle ≥ 10°), and expert evaluation. Reported diagnosis of idiopathic scoliosis/kyphoscoliosis was not part of the allocation criteria but is indicated in the diagram to determine its concordance with DEXA-identified adolescent idiopathic scoliosis. ✅: did meet criteria; ❌ did not meet criteria

To group DEXA scans as (i) “*likely AIS,*” (ii) “*without scoliosis,*” and (iii) “*uncertain to have AIS,*” both the quantitative modified Ferguson scoliosis curve measurements and qualitative expert evaluation of the DEXA scans were used (Fig. 2). Of note, a reported diagnosis of idiopathic scoliosis/kyphoscoliosis at any time from ages 10–20 years was also noted (Fig. 2), but not considered to be reliable for grouping participants as some participants reported diagnosis where scans showed no scoliosis curve. Rather, those identified as “*likely AIS*” had both a modified Ferguson angle ≥ 10°, and the expert reviewer determined that the scoliosis curves were consistent with AIS. The group “*without scoliosis*” had a modified Ferguson angle of < 10°, and the expert reviewer determined that any scoliosis curves between 8 and 10° were not consistent with AIS. The group that was “*uncertain to have AIS*” presented with either a discord between criteria for a modified Ferguson angle ≥ 10° and expert evaluation or had a reported diagnosis of AIS with neither sufficient angle or expert evaluation agreement.

### Statistical Analysis

Descriptive statistics were used to report prevalence and curve characteristics. To assess the inter-rater reliability for modified Ferguson measures from DEXA scans, statistical analysis was performed using RStudio (2020) software (version 1.3.959, PBC, Boston, MA, USA). Bland–Altman plots were used to visualize the data, and intra-class correlation (ICC) with a two-way random effects model with absolute agreement were calculated. The ICC values were interpreted as poor (< 0.40), fair (0.40–0.59), good (0.60–0.74), and excellent (0.75–1.00) [19] inter-rater agreement. To assess the consistency between the two raters, the standard error of measurement (SEM) was calculated as the standard deviation of the difference in modified Ferguson angle values between the two raters divided by the √2. The SEM was then used to determine a minimum detectable change, MDC_95_ = 1.96 × SEM × √2 (also referred to as 95% limit of agreement). The distribution of the difference scores was assessed using the Shapiro–Wilk test. The level of significance was set at *p* < 0.05.

## Results

### Inter-Rater Reliability of Modified Ferguson Method on DEXA Scans

Inter-rater reliability of the measurement of 41 DEXA scans of participants by two raters using the modified Ferguson method was good to excellent (ICC: 0.82; 95% CI: 0.71–0.89; *p* < 0.001). The difference between raters approximated a normal distribution (Shapiro–Wilk *p* = 0.28), the SEM: 2.2°; bias: 0.8°; and MDC_95_: 6.2°.

### Classification of Scoliosis and Distribution of Spinal Curve Angles

Twenty-six participants (females = 15, males = 11) were identified as *likely AIS*. Their measured curve angles as well as the side, spinal region and number of curves are detailed in Table 2. Of 1238 scans, 1139 (females = 559, males = 580) were identified as *without scoliosis*. The prevalence of AIS in this sample was 2.1% with a female/male ratio of 1.4:1. In the group with likely AIS, the mean (SD) modified Ferguson angle was 14.0 (3.5)° and ranged from 10 to 24°. Out of 142 scans that were evaluated by the expert, 116 scans were either *without scoliosis* or *uncertain to have AIS.*

**Table 2:** Gender and spinal curve descriptors of those with likely adolescent idiopathic scoliosis (*n* = 26). Angle as assessed by modified Ferguson method

Gender	Reported diagnosis	Angle (°)	Curve type	Region	Convexity	Apex
Female	Yes	12.9	Single	Lumbar	Left	L3
Female	Yes	19.7	Single	Thoracic	Right	T7
Female	No	13.3	Single	Lumbar	Left	L2
Male	No	13.5	Single	Lumbar	Left	L2
Female	No	10.3	Single	Thoracic	Right	T8
Female	Yes	13.7	Single	Thoracolumbar	Right	T12
Male	No	11.8	Single	Thoracic	Right	T7
Male	Yes	24.0	Double	Thoracic	Right	T7
Female	No	11.3	Single	Thoracolumbar	Left	T12
Female	No	13.1	Single	Thoracolumbar	Left	T12
Female	Yes	10.0	Single	Thoracolumbar	Right	T12
Female	Yes	17.0	Double	Thoracic	Right	T9
Female	Yes	18.6	Single	Lumbar	Left	L2
Male	Yes	15.5	Single	Thoracic	Left	T8
Male	No	15.9	Double	Lumbar	Left	L2
Female	No	12.2	Single	Lumbar	Left	L3
Female	Yes	19.4	Single	Lumbar	Left	L2
Female	Yes	15.0	Double	Thoracic	Right	T8
Male	Yes	10.7	Single	Lumbar	Left	L3
Male	Yes	11.5	Single	Lumbar	Right	L2
Male	No	10.2	Double	Thoracic	Left	T12
Male	No	15.4	Single	Thoracic	Right	T7
Female	Yes	11.4	Double	Lumbar	Left	L2
Male	Yes	10.7	Single	Thoracic	Right	T6
Male	Yes	13.8	Double	Thoracolumbar	Left	L1
Female	No	12.2	Single	Thoracic	Right	T9

**Fig. 3:**
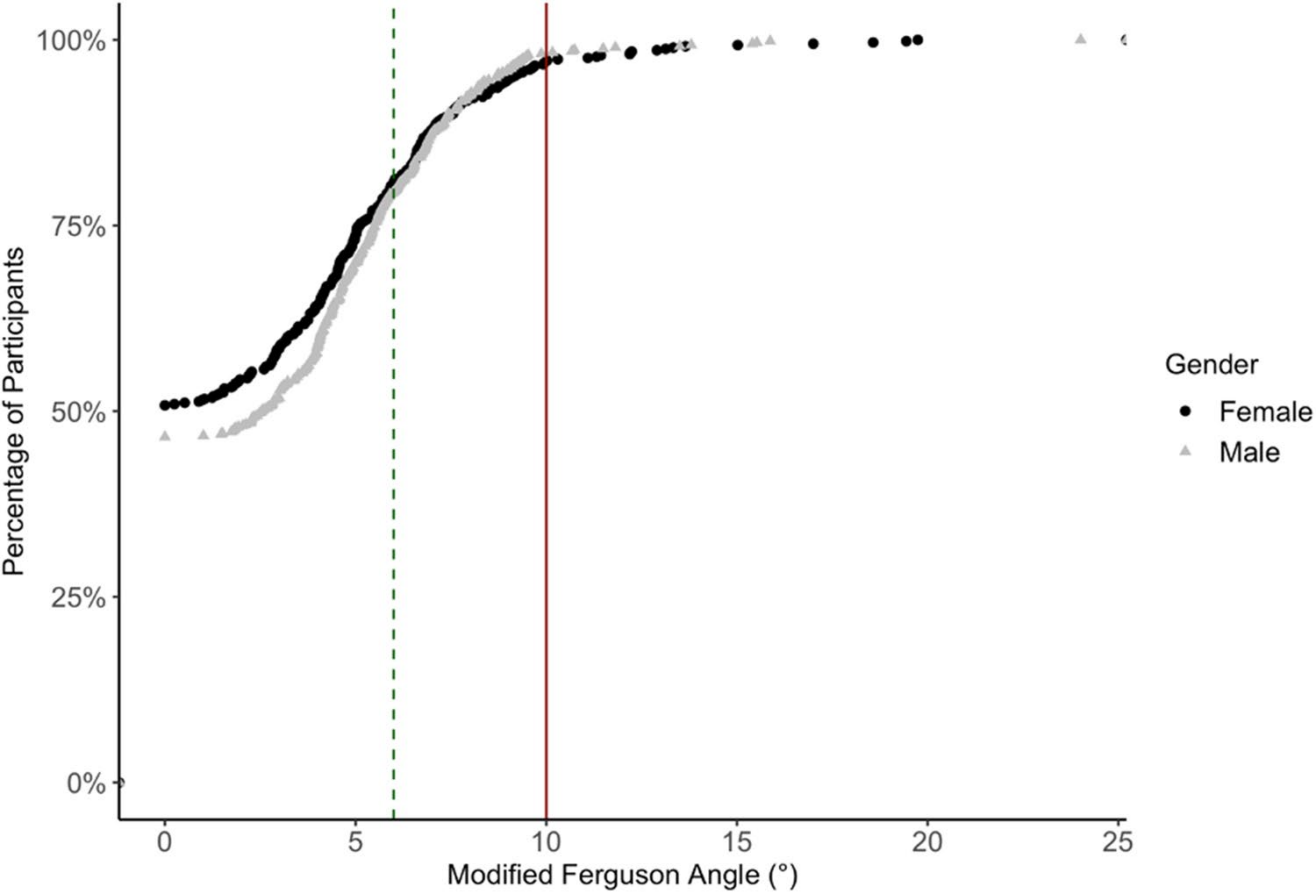
Cumulative frequency distribution of modified Ferguson angles measured in participants with *likely adolescent idiopathic scoliosis* and *without scoliosis* (female *n* = 574 and male *n* = 591). The red bold line indicates the 10° cutoff for *likely adolescent idiopathic scoliosis*, as used in the current study. The green dotted line indicates a 6° cutoff, as used in previous work [11]

The cumulative distribution frequency of the modified Ferguson angles of participants with likely AIS and *without scoliosis* is presented in Fig. 3 (total *n* = 1165). Those classified as *uncertain to have AIS* (*n* = 73) are not included in this distribution. Out of 1165 participants, 50.8% of females and 46.5% of males were assessed as having a modified Ferguson angle = 0°, and 80.8% of the females and 79.5% of the males had a modified Ferguson angle of ≤ 6°.

### Comparison of DEXA Scans Versus Reported Diagnosis

Reported diagnosis of idiopathic scoliosis/kyphoscoliosis was concordant with measured angle on DEXA ≥ 10° and expert evaluation for 15 participants (1.2%). There was notable discord between reported diagnosis and DEXA measured angle with expert evaluation as shown in Fig. 2. Taking into account the MDC_95_ of 6.2° for a 10° scoliosis curve, diagnosis of AIS was reported despite little or no scoliosis curve (i.e., DEXA < 3.8°) for 20 participants (1.6%). Eleven (0.9%) were identified as *likely AIS* on DEXA scans but did not report a diagnosis of scoliosis.

### Exclusion of Those Uncertain to have AIS

Two participants had a modified Ferguson angle of ≥ 10° and expert reviewed to have scoliosis inconsistent with AIS (curve angles 13 and 41°; male = 1, female = 1). Thirty-eight participants had a modified Ferguson angle of ≥ 10° on DEXA scan, but did not report a diagnosis of idiopathic scoliosis/kyphoscoliosis, and were evaluated by the expert DEXA review to be inconsistent with AIS (curve angles ranged from 10 to 25°; males = 29, females = 9). Thirty-two participants reported a diagnosis of idiopathic scoliosis/kyphoscoliosis but their modified Ferguson angle on DEXA scan was < 10° and were considered by the expert reviewer to have scoliosis inconsistent with AIS (curve angles ranged from 0 to 9°; males = 13, females = 19). There was one female (curve angle 9°) who reported a diagnosis of idiopathic scoliosis/kyphoscoliosis and was considered by the expert reviewer to have scoliosis consistent with AIS, but had a modified Ferguson angle < 10°.

## Discussion

Our study built on the only prior study using DEXA to identify likely AIS in a community population [11] by: (i) addressing inter-rater reliability of the Ferguson method to measure AIS curve angles, (ii) reporting the community prevalence using a combination of quantitative and qualitative scan assessment, and (iii) providing insight into the discord in identifying AIS by reported diagnosis and DEXA scans methods. Inter-rater reliability for the modified Ferguson method of measurement was good–excellent, which supports the use of this approach to measure scoliosis curve angles on DEXA scans. Expert examination of scans identified apparent curves which were likely due to issues other than AIS, including 35/142 scans potentially affected by internal organ shadowing or features of scoliosis not of idiopathic types. Using qualitative expert examination and quantitative curve angle measurements of DEXA scans, the estimated prevalence of *likely AIS* in this Australian sample at age 20 was 2.1%. The discord between measured *likely AIS* from DEXA scans and reported health professional diagnosis of AIS (ICD-9 737.3) demonstrates that that there are a proportion of adolescents who have a scoliosis curve but have not been diagnosed and suggests that some adolescents may have been misdiagnosed. Although DEXA scans are not a diagnostic tool for spinal scoliosis, when available, they enable quantitative and qualitative screening of scoliosis curves prior to formal diagnostic examination.

### DEXA to Identify Likely Idiopathic Scoliosis

The good–excellent inter-rater reliability supports the use of modified Ferguson method to measure scoliosis curve angles on DEXA. The MDC_95_ of 6.2° suggests the smallest angle which could be used to detect *likely AIS* on DEXA. This was 1.6° larger than that reported in the ALSPAC study (MDC_95_ of 4.6°) [11] with similar methods. Therefore, based on our study, to identify *likely AIS* on DEXA scans, we recommend a combination of quantitative curve angle cutoff of ≥ 10° in order to exceed the SEM (2.2°) and the MDC_95_ (6.2°), as well as using the qualitative criteria for image examination provided in the current study. The Cobb method of scoliosis measurement from digital radiographs has well-established excellent inter-rater reliability [20, 21]), with SEM (0.9°) and MDC (2.5°) [20], approximately half the magnitude of those achieved with DEXA. While this comparison supports the continued use of radiographs for accurate diagnosis and measurement of scoliosis, our results demonstrates that *likely AIS* can be identified from DEXA. Screening and identifying AIS in its early stages (i.e., prepubertal) is important as the risk of scoliosis curve progression is the greatest during rapid skeletal growth [22]. More research is needed to determine the feasibility of using DEXA as a screening tool for detecting AIS.

### DEXA to Estimate Prevalence of Idiopathic Scoliosis

The DEXA scans were able to provide an estimate of AIS in an Australian sample, at age 20 in 2009–2012. Our *likely AIS* prevalence of 2.1% with a DEXA measurement cutoff of 10° was at the lower end of the estimated global prevalence of 2–3% [1], and about two-thirds of the reported 3.1% from a cohort of ~ 3600 adolescents in South Australia, at ages 14–16, in 1982 using standing radiographs [23]. The ALSPAC study reported a prevalence of 5.9% with DEXA scans taken at age 15 using a 6° cutoff [11]. The lower cutoff angle in that study took into account that curve angles measured with Cobb method in supine radiographs resulted in ~ 7–10° smaller angles than standing radiographs [15, 24–26], and modified Ferguson curve angles on supine DEXA scans resulted in a smaller mean angle of 11.7° than modified Ferguson curve angles on standing radiographs [11]. The higher prevalence reported in that study with the 6° cutoff may represent increased sensitivity to detect scoliosis, whereas the lower prevalence reported in the current study with a 10° cutoff increased specificity of identifying *likely AIS*. The prevalence of DEXA-identified AIS was determined with 26 participants (2.1%), after excluding 73 scans that were *uncertain to have AIS*. In the excluded group that we were *uncertain to have AIS*, 35/73 participants reported a diagnosis of idiopathic scoliosis/kyphoscoliosis. If those 35 participants were included, the estimated prevalence would increase to 4.9%. This expanded prevalence rate is much greater in comparison with previously reported prevalence rates (2–3%) [1]. Despite our DEXA-based prevalence estimate being similar to the estimated global prevalence [1], studies confirming the accuracy of angle measures and expert evaluation from DEXA to determine the presence of AIS are needed to improve confidence in prevalence measurement using DEXA scans.

### Exclusion of Those Uncertain to have AIS

Out of 1238 participants, 73 were *uncertain to have AIS* and were classified in four sub-groups. Two of the sub-groups had disagreement between the measured curve angle and expert evaluation, which could represent apparent scoliosis curve angles due to organ shadowing (*n* = 38) and non-idiopathic scoliosis (*n* = 2). For the third sub-group, the measured curve angle and expert evaluation both determined that the participants did not have scoliosis, though a health professional diagnosis of idiopathic scoliosis/kyphoscoliosis had been reported (*n* = 32), which could represent participants who were falsely diagnosed with idiopathic scoliosis. The fourth sub-group (*n* = 1) was determined by the expert and a reported diagnosis of idiopathic scoliosis/kyphoscoliosis, but had a measured modified Ferguson angle of 9°, though it is possible that a larger angle may have been present if measured by standing radiograph and Cobb angle. We lacked any further clinical information to confirm diagnosis of idiopathic scoliosis. It is possible that those we were *uncertain to have AIS* may contain some people with AIS, but in the absence of any further clinical information, the specificity of screening for *likely AIS* was prioritized over sensitivity for this study. Future research could examine the sensitivity and specificity of the quantitative curve angle measurement on DEXA scans and expert qualitative evaluation against confirmed clinical diagnoses based on standing radiographs (the current gold standard for diagnosis of AIS).

### Concordance of Health Professional Diagnosis of Idiopathic Scoliosis/Kyphoscoliosis and Likely AIS Identified on DEXA

A unique contribution of the Raine Study was the ability to explore the concordance between two methods of identifying AIS within the cohort: reported health professional diagnosis of idiopathic scoliosis/kyphoscoliosis and *likely AIS* identified on DEXA. For the Raine cohort, there was no information about the health professional who diagnosed the condition or diagnostic method used. Of the 50 people who first reported diagnosis of AIS from age 10 or older, 20 had little or no scoliosis curve visible on DEXA. False positive diagnosis raises the possibility of low-value use of health system resources, including unnecessary consultations, spinal imaging and interventions, and risk of psychosocial stress [27]. Of the 1238 people with DEXA scans, 11 had DEXA curve angles ≥ 10° but did not report any diagnosis of scoliosis. Failure to identify AIS may result in insufficient care of the scoliosis [28], and late detection of AIS may lead to increased need for instrumented spinal fusion surgery [9]. In Australia, there has been no formal school screening for the presence of scoliosis since the 1990s, with AIS identification only supported through public awareness campaigns suggesting concerned people visit a health practitioner for an assessment [8]. Further research is needed to determine whether a failure to diagnose AIS during adolescence contributes to adverse health impacts and costs in adulthood, and to identify the health-related impact and cost of false positive diagnosis of AIS.

Limitations in this study included the low-resolution images from 2009 to 2012, compared to images from contemporary scanners which could enable more accurate curve angle measurement. The sample size of those identified with *likely AIS* was modest (*n* = 26). A larger sample size of participants with *likely AIS* would allow greater confidence in the distribution of modified Ferguson angles and curve characteristics representative of the wider population with AIS. Despite these limitations, the Raine Study is a prospective longitudinal cohort that provided a uniquely unbiased sample to estimate population prevalence and explore curve characteristics of scoliosis. Future exploration of data associated with the groups identified in this study could provide insights toward factors that contribute to AIS as well as the clinical impacts and costs associated with AIS.

## Conclusion

The good–excellent inter-rater reliability supports the use of modified Ferguson method to measure scoliosis curve angles on DEXA. This study showed the potential utility of using a combination of quantitative measurement and qualitative criteria to evaluate DEXA images, to identify *likely AIS* for reporting prevalence. Without formal school screening, the analysis of DEXA in this population sample suggested that relying on current health professional diagnosis alone could result in 2.5% of this cohort being at risk of false positive diagnosis or delay in necessary management due to non-diagnosis of AIS.

## Supplementary Information

Below is the link to the electronic supplementary material.

Supplementary file1 (PDF 746 KB)

## Data Availability

The data used to generate the results in this publication are available upon request as ethical restrictions exist, and data were obtained from a third party. Readers and interested researchers may contact the Executive of the Raine Study to request the data.
