# Supplementary material for: Advancing Use of DEXA Scans to Quantitatively and Qualitatively Evaluate Lateral Spinal Curves, for Preliminary Identification of Adolescent Idiopathic Scoliosis
Source: Calcif Tissue Int. 2023 Mar 13;112(6):656–65. doi: 10.1007/s00223-023-01075-2 (PMC10198858; doi:10.1007/s00223-023-01075-2)
Supplement: Supplementary file 1 — Supplementary file1 (PDF 746 KB) [file 223_2023_1075_MOESM1_ESM.pdf]

Advancing use of DEXA scans to quantitatively and qualitatively evaluate lateral spinal curves, for preliminary identification of adolescent idiopathic scoliosis

Ng, PTT[1,2], Straker, L[3], Tucker, K[1], Izatt, MT[4], and Claus, A[5,6]

1 The University of Queensland, Laboratory for Motor Control and Pain Research, School of Biomedical Sciences, QLD, Australia

2 KK Women's and Children's Hospital, Physiotherapy Department, Singapore, Singapore.

3 Curtin University, School of Allied Health, WA, Australia

4 Queensland University of Technology at the Centre for Children's Health Research, Biomechanics and Spine Research Group, QLD, Australia

5 The University of Queensland, School of Health & Rehabilitation Sciences, QLD, Australia

6 Royal Brisbane and Women's Hospital, Tess Cramond Pain and Research Centre, QLD, Australia

Corresponding Author: Phoebe Ng

phoebe.ng@uqconnect.edu.au

Fig A1 Example image of: poor positioning the DEXA scanner with lateral translation of shoulders relative to the pelvis.

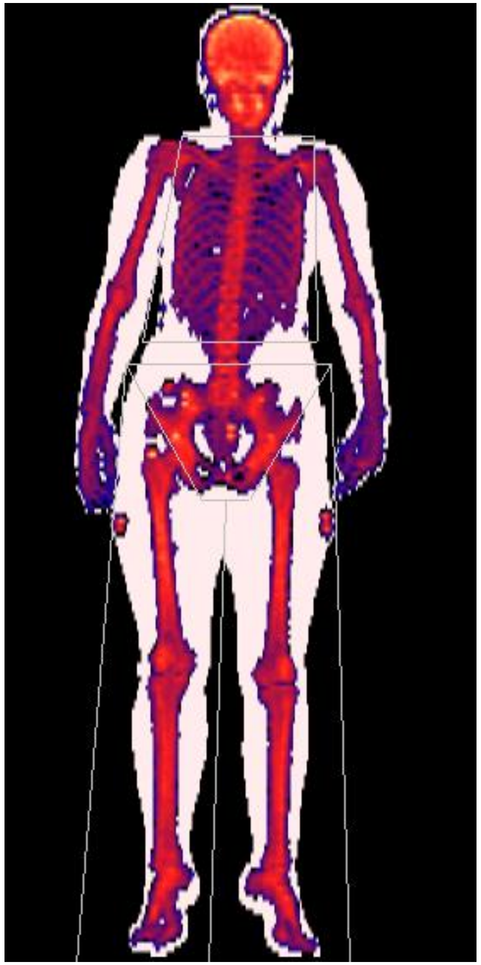

Fig A2 Example image of shadowing of the internal organs in mid thoracic spine due to aortic arch giving rise to a false impression of thoracic scoliosis.

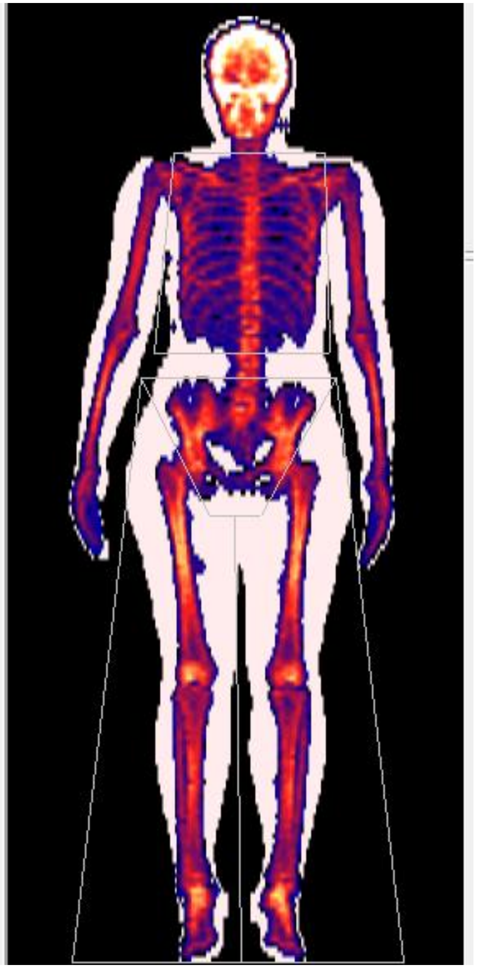

Fig A3 Example image of other types of scoliosis likely not of idiopathic type due to acutely angulated spine over 2 segments in mid lumbar spine.

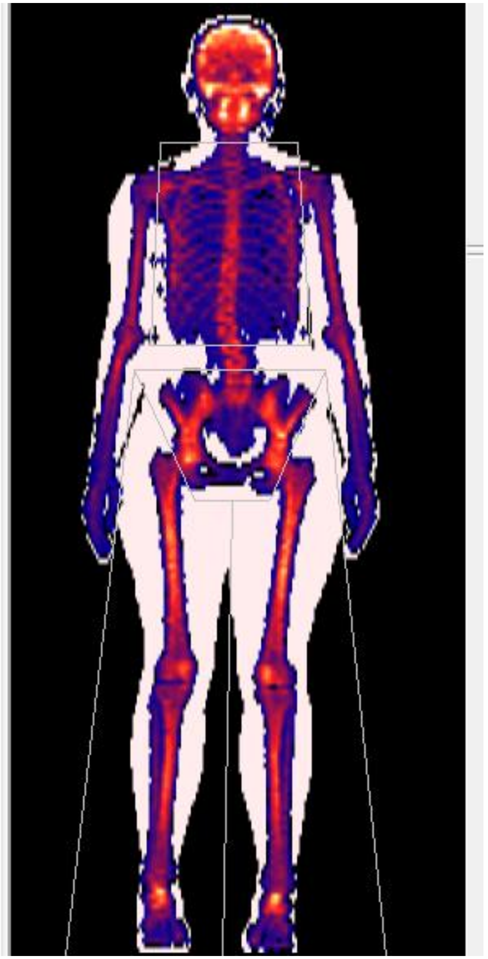

Fig A4 All images of participants with likely adolescent idiopathic scoliosis.

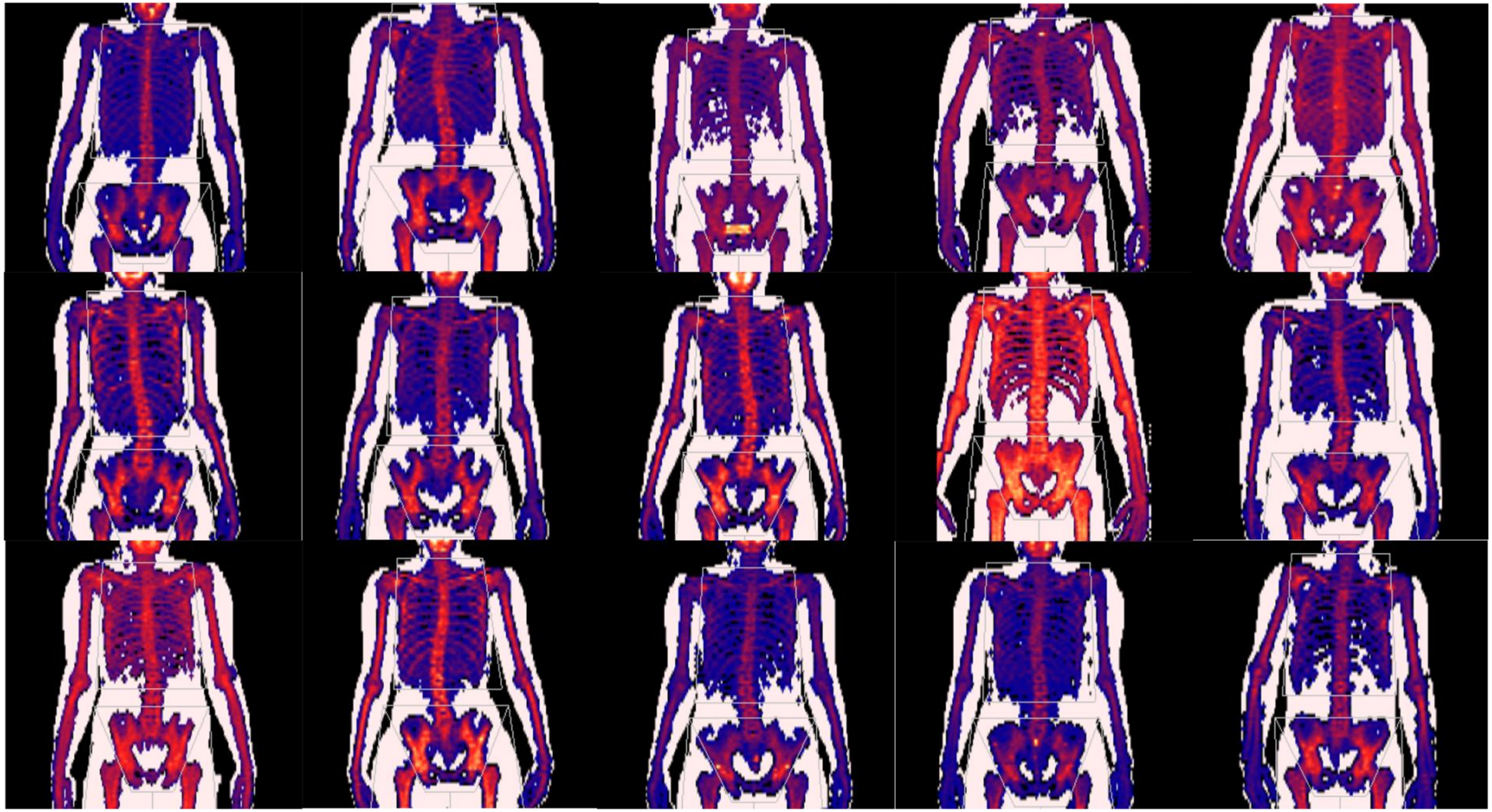

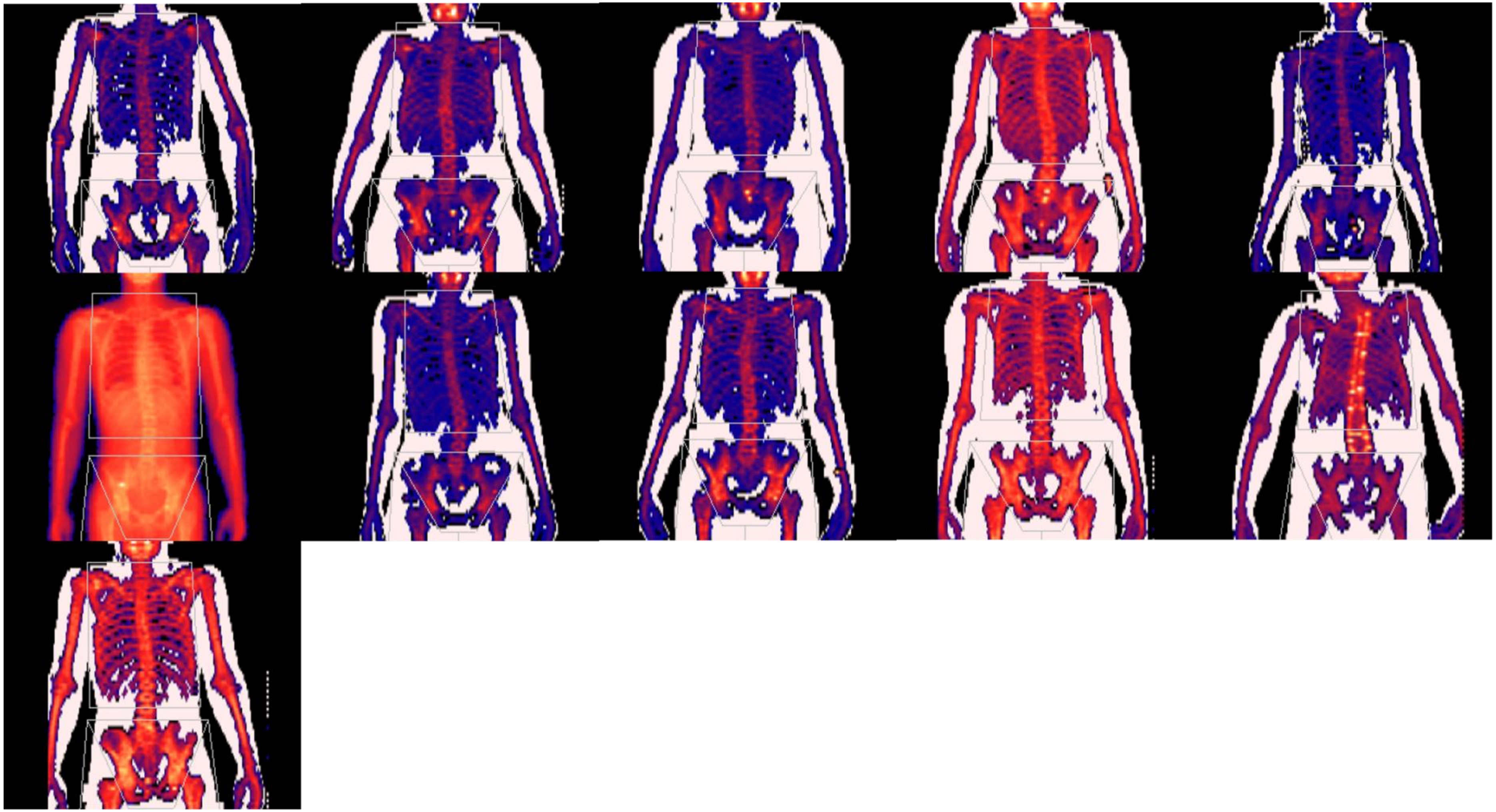

The image on the far right of the second last row demonstrates a participant with surgical spinal instrumentation in adolescent idiopathic scoliosis. Colours of the scans vary depending on the contrast of the downloaded digital image.
